# From Bioink to Tissue: Exploring Chitosan-Agarose Composite in the Context of Printability and Cellular Behaviour

**DOI:** 10.3390/molecules29194648

**Published:** 2024-09-30

**Authors:** Szymon Mania, Adrianna Banach-Kopeć, Natalia Maciejewska, Katarzyna Czerwiec, Paulina Słonimska, Milena Deptuła, Jakub Baczyński-Keller, Michał Pikuła, Paweł Sachadyn, Robert Tylingo

**Affiliations:** 1 Department of Chemistry, Technology and Biotechnology of Food, Faculty of Chemistry, Gdańsk University of Technology, 80-233 Gdańsk, Poland; szymon.mania@pg.edu.pl (S.M.); robertt@pg.edu.pl (R.T.); 2 Department of Pharmaceutical Technology and Biochemistry, Gdańsk University of Technology, 80-233 Gdańsk, Poland; natalia.maciejewska@pg.edu.pl; 3 Division of Clinical Anatomy, Medical University of Gdańsk, 80-210 Gdańsk, Poland; katarzyna.czerwiec@gumed.edu.pl; 4 Laboratory for Regenerative Biotechnology, Gdańsk University of Technology, 80-233 Gdańsk, Poland; paulina.slonimska@pg.edu.pl (P.S.); jakub.keller@pg.edu.pl (J.B.-K.); psach@pg.edu.pl (P.S.); 5 Laboratory of Tissue Engineering and Regenerative Medicine, Division of Embryology, Medical University of Gdańsk, 80-210 Gdańsk, Poland; milena.deptula@gumed.edu.pl (M.D.); michal.pikula@gumed.edu.pl (M.P.)

**Keywords:** chitosan, agarose, bioprinting, natural polymers, tissue engineering, biocompatibility

## Abstract

This study presents an innovative method for producing thermosensitive bioink from chitosan hydrogels saturated with carbon dioxide and agarose. It focuses on a detailed characterisation of their physicochemical properties and potential applications in biomedicine and tissue engineering. The ORO test approved the rapid regeneration of the three-dimensional structure of chitosan–agarose composites in a unidirectional bench press simulation test. The diffusion of dyes through the chitosan–agarose hydrogel membranes strongly depended on the share of both polymers in the composite and the molecular weight of the dyes. Glucose, as a nutrient marker, also diffused through all membranes regardless of composition. Biocompatibility assessment using MTT tests on 46BR.1N fibroblasts and HaCaT keratinocytes confirmed the safety of the bioink. The regenerative potential of the bioink was confirmed by efficient cell migration, especially HaCaT. Long-term viability studies showed that chitosan–agarose scaffolds, unlike the agarose ones, support cell proliferation and survival, especially 14 days after bioink extrusion. Experiments in a skin wound model in mice confirmed the biocompatibility of the tested dressing and the beneficial action of chitosan on healing. Studies on vessel formation in chicken embryos highlight the potential of the chitosan–agarose composition to enhance proangiogenic effects. This composition meets all entry criteria and possesses excellent biological properties.

## 1. Introduction

Bioprinting is an excellent tool for the production and study of artificial tissues, and is thus a potential tool in skin regeneration [1]. Compared to typical skin regeneration methods, bioprinting offers better automation and standardisation for clinical uses and accuracy in incorporating living cells, growth factors, and other biomolecules [2]. Generally, the main advantage of bioprinting over other techniques is the ability to print not only scaffolds of a specific, personalised shape but also a properly designed internal network in which all processes necessary for regeneration and tissue or organ formation occur.

However, one of its major challenges remains the development of bioink and the application of the appropriate crosslinking technique, which will allow obtaining an object with specific physicochemical and biological properties. Such a bioink must be printable, i.e., have appropriate rheological parameters, and the obtained scaffolds must be mechanically stable and biocompatible, i.e., provide an appropriate environment in which cells will survive, proliferate, migrate, and differentiate, forming tissues [3,4,5]. These three parameters determine the quality of the bioink, which, as it turns out, are contradictory because the higher the viscosity of the bioink, the better the printability, and conversely, the worse the biocompatibility. Therefore, already at the stage of designing the bioink and determining its application, it is necessary to focus research and find a compromise between these properties [6].

An example of systems that seem to possess both good physical properties and are biocompatible are hydrogels, which form a three-dimensional network of hydrophilic polymer chains. Additionally, hydrogels can effectively recreate the extracellular matrix and the natural environment of cells. They also have mechanical properties similar to tissues and can provide cells with structural support and hydration. Another desirable property of hydrogels is shear-thinning, which allows the extrusion of bioinks under high shear stress while maintaining the mechanical properties of the system [7].

In tissue engineering and bioprinting, both natural polymers, such as alginate, chitosan (CS), hyaluronic acid, and gelatin, and synthetic ones, which include polyacrylamide, polyvinyl alcohol, polyethylene glycol, and polylactic acid, are used. The main advantages of natural polymers are their biocompatibility and specific enzymatic degradation, as well as the presence of functional groups and domains, which provide better interactions with cells than synthetic ones. These, in turn, are characterised by strong water adsorption and high gel strength. However, despite the introduction of synthetic polymers, which allow researchers’ precise control of the gels’ structure and properties, special attention should be paid to their biocompatibility. This problem concerns potential residues during the production of such polymers, i.e., unreacted monomers and remnants of initiators [8].

This work is a continuation of a series of studies on the development of a hydrogel polymer bioink based on CS obtained by the carbon dioxide saturation method and agarose (AG). The concept of combining CS in bioink with fibroblast and keratinocyte stem cells emerges from the positive impact of this polymer on the skin tissue regeneration process, confirmed by numerous studies. The primary mechanism of action of CS is based on the activation of key components of the immune system, namely inflammatory cells, macrophages, and fibroblasts. This, in turn, contributes to the shortening of the inflammatory phase and accelerates the transition to the proliferation phase, which is essential in rebuilding damaged tissue. Furthermore, CS promotes the formation of granulation tissue and angiogenic processes, facilitating the effective deposition of collagen fibres, which is crucial for the repair of skin damage [9,10]. Angiogenesis plays a crucial role in regeneration, as vascularisation is needed to supply oxygen, nutrients, and growth factors to the regenerating tissue. Proper angiogenesis can accelerate wound healing and prevent necrosis; however, abnormal angiogenesis contributes to a significant slowdown of this process [11].

The main issue with current bioprinting formulations lies in their limited versatility. Many commercially available and researched systems rely on three primary polymers: alginate, collagen, and gelatin. These systems have gained widespread adoption due to their simplicity in crosslinking using methods like calcium chloride and UV radiation, especially after modification to methacrylated derivatives. However, these formulations predominantly focus on supporting cell viability post-printing, lacking additional biological effects such as promoting angiogenesis or cellular differentiation. To date, only a few solutions for alternative bioink compositions have been reported in the literature. One approach involves carboxymethylchitosan combined with agarose and sodium alginate [6], while another utilises N,O-carboxymethylchitosan with agarose [12]. Modifications to chitosan to enhance solubility aim to eliminate the use of cytotoxic acids but necessitate the use of organic solvents for derivatisation. Nevertheless, chemical modifications of polymers pose risks of residual toxic solvents and involve time-consuming processes, complicating their practical application and potentially leading to the release of cytotoxic degradation products over time.

This study aims to demonstrate additional physicochemical and biological properties confirming the practical application and meeting the acceptance criteria of a chitosan–agarose bioink, essential for biomaterials in tissue engineering. Our previous research has demonstrated that blending two polymers with diverse physicochemical and biological properties enables the creation of a bioink that fulfils all necessary system requirements: it is printable, is biocompatible, and possesses suitable rheological properties for bioprinting applications [13]. We have identified composites with significant application potential through rheological studies and extrusion tests. These blends underwent rigorous biological testing to assess their suitability for wound healing and broader tissue engineering applications. This approach represents a straightforward solution for producing a bioink, based on just two components, that already meets numerous critical criteria for tissue engineering applications from inception.

## 2. Results

### 2.1. Physiochemical Properties of Chitosan–Agarose Composition

#### 2.1.1. Rheological Properties

**Figure 1:**
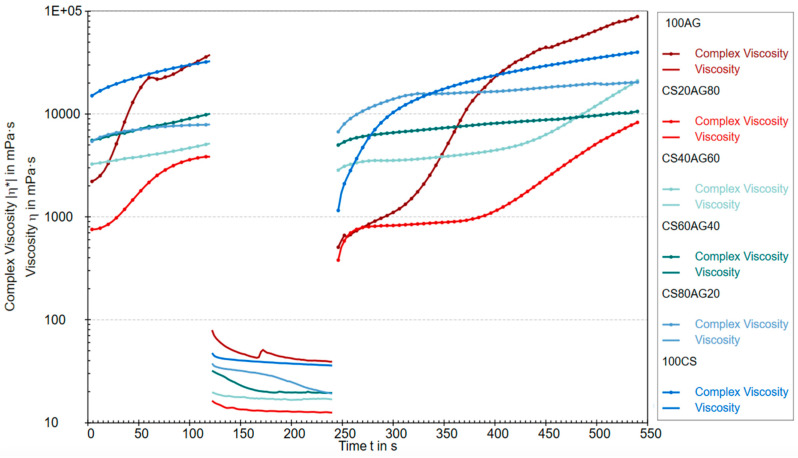
Comparison of complex viscosity and dynamic viscosity during the ORO test.

The rheological properties of CS agarose solutions and their composites were evaluated using the three-interval thixotropy test (3ITT), conducted in the oscillatory–rotational–oscillatory (ORO) variant, which applies high rotational stress immediately after a low-strain oscillatory stage within the LVE region. This approach effectively simulates the unidirectional extrusion of bioink from the printer head and assesses its behaviour post-stress relaxation under conditions similar to the physiological ones optimal for cells in the prepared bioink for printing (37 °C) [14]. Figure 1 shows the change in complex viscosity (oscillatory part) and dynamic viscosity (rotational part) over time for 1% solutions of CS and AG, as well as their mixtures. The viscosity changes over time suggest processes related to the degradation and regeneration of the studied fluids’ structure. Complex viscosity describes a material’s resistance to deformation under stress, encompassing both viscous (loss) and elastic components, making it particularly useful for analysing thixotropic materials with time-dependent structures. The viscosity levels in the first test cycle indicate that chitosan and agarose as single-component solutions exhibit a more developed structure that is resistant to deformation under dynamic loads. In other words, under oscillatory conditions in the LVE region, the internal molecular connections in these single-component materials are more robust than in their composites. Introducing rapid rotation in the second region caused the destruction of the structure of all tested samples. Viscosity values in this range confirm the trend observed in the first cycle, where a chitosan content of 20% and 40% in the composite shows the most significant susceptibility to deformation induced by stresses mimicking unidirectional shear. The third region of the graph indicates the ability to rebuild the structure of the studied polymer sols, but with different characteristics. Based on this part of the graph, it can be concluded that the reconstruction of the structure of pure agarose takes the longest, but at the same time, the most durable network is created. The values of the complex viscosity of composite materials at the end of the first oscillation and at the beginning of the third are similar, which indicates that the three-dimensional structure of the material will be quickly recovered after unidirectional squeezing from the syringe, which is an advantage of using the composite.

#### 2.1.2. Permeability

The permeability of scaffolds to nutrients, gases, and metabolites is a key feature that provides the scaffold-embedded cells with the correct conditions for proliferation, differentiation, and migration. For this purpose, a series of measurements were carried out to determine how changing the composition of the scaffold, i.e., the ratio of AG to CS, affects the migration of components of the standard glucose and compounds differing in molecular weight.

**Figure 2:**
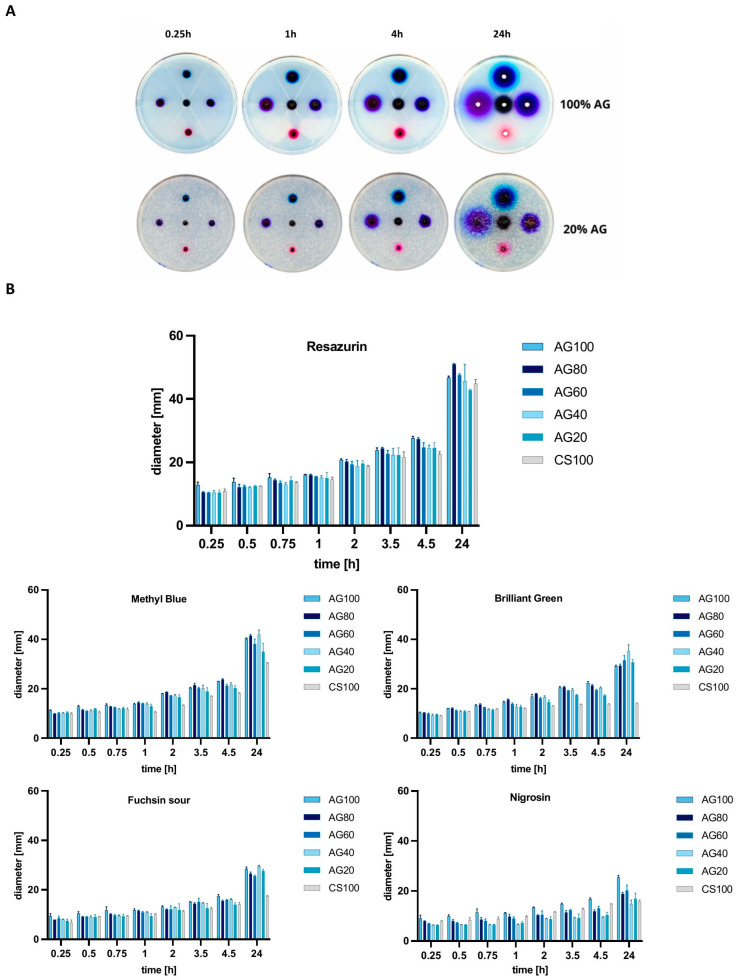
(**A**) Images of dye-applied hydrogel samples over 24 h of incubation. (**B**) Sizes of dye stain diameters from 0.25 to 24 h at specific time points. Data represent mean ± standard deviation; *p* < 0.05.

This study of the effect of the molecular weight of dyes on their permeability through membranes showed that the diffusion rate for the compounds with the lowest molecular masses is the highest (Figure 2). Furthermore, for the dye with the lowest molecular weight, Resazurin (251.17 g/mol), the most prominent spot diameters were observed, indicating its highest permeability through hydrogel membranes, more than double compared to Nigrosin (616.49 g/mol). For example, increasing the molecular weight of a compound from approximately 251 to 482 significantly reduced permeability, which after 24 h of incubation for Resazurin was about 50 mm, and for Brilliant Green, it was below 40 mm in dye spot diameter. Comparing Nigrosin and Resazurin dye diffusion, for the 100% AG system, the average dye stain after 24 h was approximately 1.6 times smaller.

Based on the conducted studies, it can be concluded that the composition of CS/AG composites, i.e., the proportion of each polymer and the molecular weight of diffusing substances, significantly impacts their permeability through hydrogel membranes. It has been shown that 100% AG membranes display a higher permeability than 100% CS membranes. This phenomenon is consistent with the results available in the scientific literature, which explain it by the lower crosslinking density and higher porosity of AG membranes. The looser structure of AG facilitates the diffusion of particles. On the other hand, adding CS to the composite reduces the diffusion of dyes through the membrane, mainly observed as the molecular weight of the tested substance increases. This phenomenon can be attributed to the stronger intermolecular bonds of CS affecting, in turn, its lower permeability. Consequently, mixing CS with AG makes it possible to regulate the porosity and crosslinking density of membranes, allowing for the modification of their permeability.

Also, Reys et al. deduced that pure AG shows better permeability towards methylene blue than a fucoidan–agarose-based hydrogel. As a result of the more robust interactions between the polymers, a more cohesive and compact system is formed, resulting in slower diffusion of the dye. Furthermore, the same study showed that permeability decreases with increasing agarose concentration, resulting in a denser hydrogel matrix [15]. Based on these results, it can be assumed that compounds with a lower molecular weight, such as glucose (180.17 g/mol), will effectively diffuse through hydrogel membranes, regardless of the CS/AG composition. Such membrane properties open up possibilities for their application in many fields, from biotechnology to regenerative medicine, where controlling substance diffusion through membranes is crucial.

**Figure 3:**
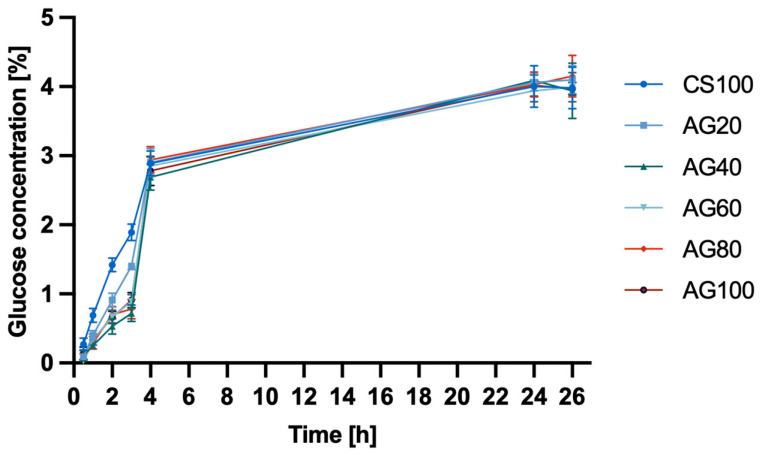
Glucose diffusion into the acceptor chamber through chitosan CS, agarose AG, and chitosan–agarose CS/AG membranes at 25 °C.

Therefore, in the study of glucose diffusion through hydrogel membranes (Figure 3) based on CS, CS/AG, and AG, no significant differences were observed in the change in glucose concentration in the acceptor chamber over time. Initially, the CS membrane demonstrated a greater ability to diffuse glucose molecules. However, after 4 h, the glucose concentration diffusing through the hydrogel membranes became similar. After 24 h, the glucose concentrations in both the acceptor and donor chambers equalised, with the glucose concentration after 24 h being 3.97 ± 0.2.

### 2.2. Biological Properties of Chitosan–Agarose Composition

#### 2.2.1. MTT Test

The MTT assay was conducted on skin cell lines, namely fibroblasts 46BR.1N and keratinocytes HaCaT, for which the developed bioinks will be used as carriers in future applications. The results provide further evidence confirming the biological safety of CS, AG, and their composites, and they are consistent with our previous studies, which, according to ISO standard 10993-5, demonstrated that both AG and CS of various degrees of DD and MW are not cytotoxic towards the standardised mouse fibroblast L929 cell line [13]. In the present study, in order to investigate potential cytotoxicity towards skin cells, cell viability was assessed following 24 h contact with the extracts from the tested samples.

**Figure 4:**
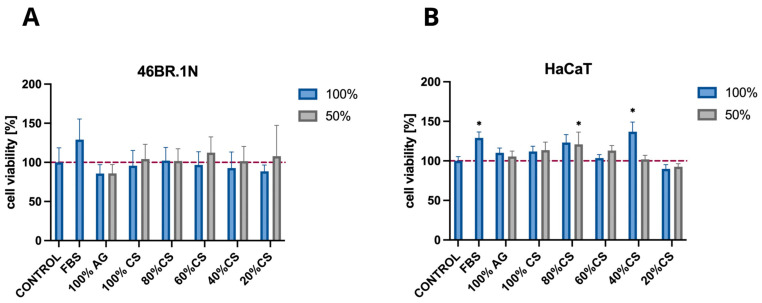
The cytotoxicity of chitosan (CS), agarose (AG), and their composites CS/AG following 24 h incubation in two concentrations against (**A**) fibroblasts 46BR.1N and (**B**) keratinocytes HaCaT. Data are expressed as the mean ± standard deviation of three independent experiments. The results were analysed by one-way ANOVA with comparisons vs. control: *p* > 0.05, * *p* < 0.05.

According to ISO standard 10993-5, materials for which cell viability exceeds 70% are considered biocompatible. The tested samples showed high cell viability, with values ranging from approximately 85 to 112% (Figure 4A) for the 46BR.1N cell line and from approximately 90 to 137% (Figure 4B) for the HaCaT line, suggesting the favourable biological safety of these materials.

#### 2.2.2. Migration Test

An essential aspect of cellular scaffold design is the selection of biomaterials capable of stimulating cell migration processes. This applies to both cells contained in the bioink and those naturally present at the site of the scaffold application. Currently, two approaches are used in bioprinting: the first involves placing cells in the bioink, within which they are supposed to differentiate and migrate; the second promotes the migration of cells from the body into the scaffold.

The use of extracts in the Ibidi wound healing assay model was due to the need to assess the impact of biomaterials under conditions close to real applications, where, in addition to placing cells inside the scaffolds, which are porous, there is also a necessity for cell migration into the pores where they proliferate and differentiate. The fact that extracts were used also stemmed from the fact that AG as well as CS/AG systems gel at 37 °C, as do CS solutions along with carbon dioxide release, especially at elevated temperatures. Consequently, examining cell migration using the above set directly in solutions would have been impossible. Furthermore, CS, AG, and CS/AG solutions have different viscosities before and after gelation, significantly disturbing the measurement. Therefore, the effect on cell migration and the absence of cytotoxicity were assessed by determining cell viability using extracts obtained from the tested biomaterials.

**Figure 5:**
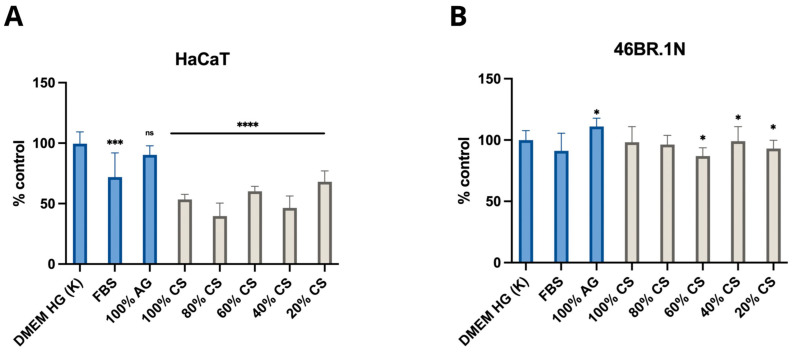
Effects of MMW chitosan (CS), agarose (AG), and their composites on the migration of (**A**) fibroblasts 46BR.1N and (**B**) keratinocytes HaCaT. The graphs show the results (calculated from scratch area) of at least three experiments, which are presented as the mean  ±  SEM. The results were analysed by one-way ANOVA with comparison to control DMEM HG. ns (not statistically significant, *p* > 0.05), * *p* < 0.05, *** *p* < 0.001, **** *p* < 0.0001).

The analysis of the results showed (Figure 5) that in the case of HaCaT cells, all CS extracts significantly supported cell migration, achieving better results than the negative (cells stimulated with DMEM HG medium) and positive (cells stimulated with DMEM HG medium containing 10% FBS) controls. In the case of AG, no statistically significant differences were observed compared to the control. For the 46BR.1N cells, despite statistically significant differences compared to the control, the pro-migratory effect was moderate, and the lack of stimulation by FBS suggests the specific requirements of these cells. These results indicate that AG extracts do not support cell migration, while CS shows promising pro-migratory properties for HaCaT cells, which is not observed in cultures of 46BR.1N fibroblasts.

#### 2.2.3. Cell Viability

As part of the further analysis of the biological properties of the tested bioinks, the cell viability of the HaCaT line and 46BR.1N fibroblasts in contact with scaffolds containing 1% AG and the CS/AG composite in a 1:1 ratio was examined. The viability assessment was conducted at three time points, 2, 7, and 14 days post-scaffold extrusion, allowing for the observation of the long-term effects of the tested biomaterials on the cells. This selection of time points allowed for the examination of both the biological safety of the system, i.e., its cytotoxic effects, and its influence on cell proliferation and migration.

**Figure 6:**
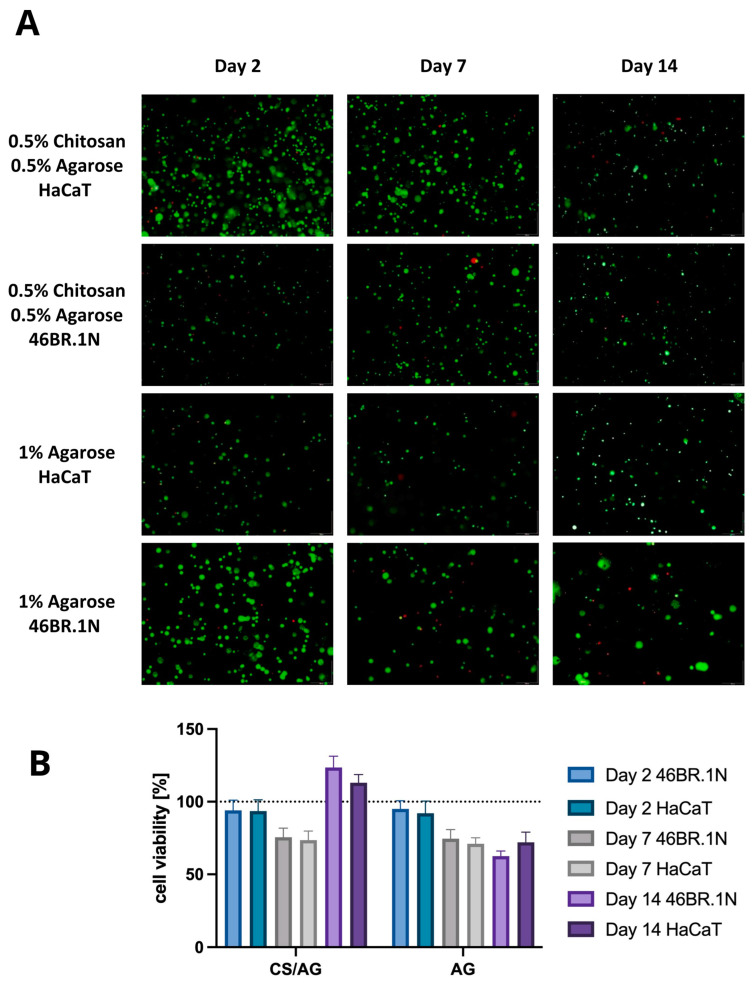
(**A**) Viability of 46BR.1N fibroblasts and HaCaT keratinocytes embedded in chitosan–agarose CS/AG composite and in agarose AG alone. This figure presents fluorescent microscope images showing live–dead staining of cells in the scaffolds on days 2, 7, and 14 of cultivation. Viable cells are stained green with calcein, while dead cells are stained red with propidium iodide; the scale bar is 250 µm. (**B**) Graph of changes in survival rates of both cell lines over time. The results were evaluated using a Leica DMIL LED inverted microscope and Graphpad Prism 5.0.

According to the results presented in Figure 6, after two days of incubation, a similar viability was observed for both cell lines across all tested systems, oscillating around 100%. These results indicate a lack of cytotoxicity, previously demonstrated in the MTT assays for the extracts of the tested biomaterials. Seven days after scaffold extrusion, a slight decrease in the viability of both cell lines and in both types of scaffolds was observed; however, these values remained at an acceptable level, ranging between 70% and 78%. This decrease in living cells may be attributed to adapting to the new environment. Nevertheless, a positive impact on the survival and proliferation of cells was noted for the CS/AG composite after 14 days for both 46BR.1N and HaCaT cells, where viability reached 117% and 129%, respectively. In systems based solely on AG, cells tended to cluster outside the scaffold area, resulting in a slight decrease in cell viability within the scaffold. Moreover, this observation aligns with the literature data indicating that AG scaffolds do not promote cell adhesion.

In summary, both materials tested demonstrated compatibility with HaCaT and 46BR.1N cells, showing no cytotoxic action over up to 14 days of incubation. Furthermore, the data confirm that the CS/AG composite is not only compatible with the cells but also promotes their proliferation, providing favourable rheological parameters while maintaining high cell viability.

The bioprinting process significantly impacts cell survival, especially during extrusion, influenced by factors like bioink viscosity, printing speed, pressure, nozzle geometry, build platform temperature, needle diameter, and construct geometry [16]. Insufficient initial cell density can lead to slower cell and tissue growth rates and inadequate scaffold integration in vivo [17]. The bioink’s composition, construct geometry (porosity, permeability, degradability), growth factors, peptide sequences, and culture conditions (temperature, nutrient composition) further affect cell survival [13]. Comparing cell survival rates over different time intervals is challenging due to numerous variables in bioprinting parameters. For instance, human keratinocytes and fibroblast cells in a genipin-crosslinked CS-PEG system showed up to 85% survival after 7 days [18]. In a self-crosslinkable CS–gallic acid system, NIH 3T3 cell survival was 92% after 7 days [19]. Over a more extended period, NIH 3T3 cells in an aldehyde hyaluronic acid-N-carboxymethyl chitosan–gelatin–alginate system maintained approximately 91% viability after 29 days [20]. Fluorescent staining of fibroblast and keratinocyte cells in CS/AG and AG systems revealed nearly 100% survival, indicating that the optimal extrusion temperature did not induce cell death. However, survival declined to 70% by day 7, potentially due to new environmental or mechanical stress from the printing process. Beyond this adaptation phase, survival increased to 110% by day 14, suggesting the cells adapted and began proliferating, potentially facilitated by biocompatible materials like CS supporting tissue regeneration and creating favourable conditions for cell expansion.

#### 2.2.4. Angiogenesis

**Figure 7:**
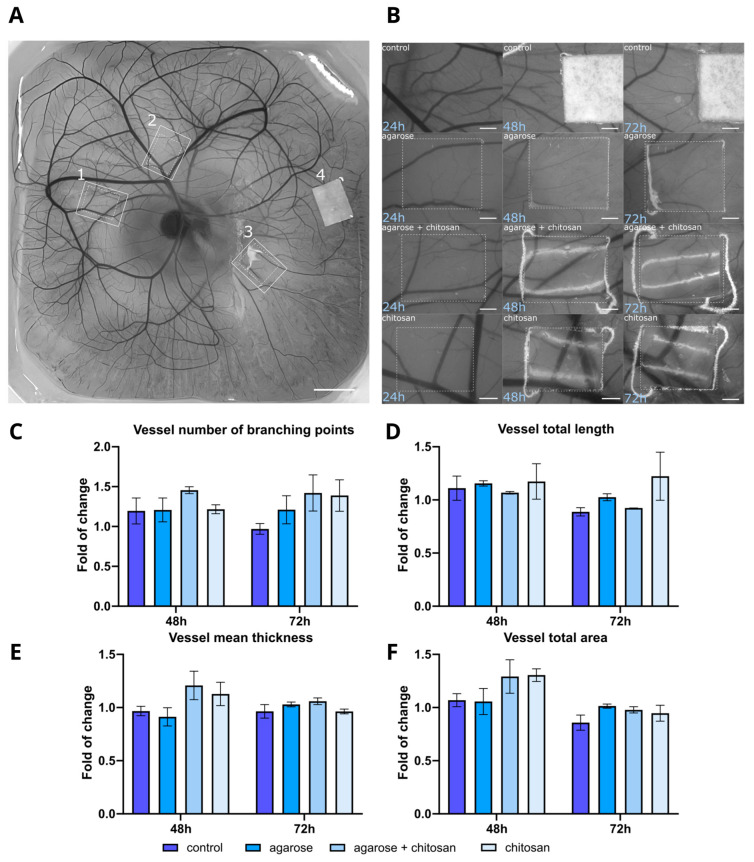
The ex ovo CAM angiogenesis assay. (**A**) Representative image of an ex ovo-cultivated chicken embryo with different tested materials on embryonic development day (EDD) 9. The materials are indicated as follows: 1—agarose + chitosan, 2—chitosan, 3—agarose, 4—Whatman filter paper. Scale bar = 5 mm. (**B**) Representative microscopic images showing material-induced angiogenesis after 24, 48, and 72 h of incubation with the materials. Scale bar = 1 mm. Analysis of changes in the vascular network, including the number of vessel branching points (**C**), total vessel length (**D**), mean vessel thickness (**E**), and total vascular area (**F**), presented as fold changes relative to the 24 h incubation. Data represent mean values  ±  s.d. from at least two independent experiments.

The proangiogenic potential of CS- and AG-based materials was evaluated using a CAM assay on 8-day-old embryos. Materials including CS, AG, and a combination of both were tested against a Whatman filter paper control (Figure 7A,B). The analysis focused on several parameters of the vascular network, including the number of branching points, vessel network length, total vascular area, and mean vessel thickness. Branching points are crucial for evaluating angiogenesis, as new vascular branches arise through mechanisms like sprouting or intussusception from existing vessels. A notable increase in branching points was observed with CS and the CS/AG combination, particularly at 72 h, while AG alone showed a slight increase compared to the control (Figure 7C). Another vital phase in angiogenesis is the extension of blood vessels. At 48 h, the total vessel length was similar across the control, AG, and CS/AG combination treatments. However, CS alone exhibited a consistent increase in vessel length at both 48 and 72 h (Figure 7D). The total vascular area reflecting the surface area of all vessels in an image increased as the vascular network expanded. As shown in Figure 7E, the total vascular area displays a slight increase across all materials over time, and it is more pronounced after 48 h, whereas after the next 24 h, it remains almost constant. The thickness of the blood vessels also influences the total vascular area. Since new vascular sprouts that form during angiogenesis are thin, they may not significantly affect the total vascular area compared to other parameters. Alterations in the average thickness of the vasculature were observed, as newly formed thin sprouts contributed to a reduction in the overall diameter of the vascular network. The mean thickness increased only with the combination of CS/AG and with CS alone at 48 h, whereas at 72 h, the mean vessel thickness remained relatively consistent across all treatments with slight variations (Figure 7F).

Although the CAM model is a recommended test for assessing the biocompatibility of biomaterials, few studies have focused on this aspect. The angiogenesis results for CS- and AG-based materials can be compared with those of the other biomaterials that were analysed in the study by Kohli et al. [21] using the CAM test. In this study, a variety of materials, natural and synthetic, and their combinations were compared. A pronounced proangiogenic effect was observed in fibrin-based materials, which significantly increased vessel density and the number of branching points compared to natural materials, such as collagen and elastin, and synthetic materials, such as PCL (poly-ε-caprolactone), which exhibited much weaker proangiogenic effects. Concerning the results presented by the research team, collagen and elastin demonstrated effects similar to CS and AG in terms of vessel density and total vascular area. Additionally, they also noted that not only the composition of biomaterials but also their porosity influences vessel growth. Biomaterials with greater porosity, such as fibrin, promoted more intense vessel growth, which may result from better access for cells and vessels to the biomaterial. Even though synthetic materials, such as PCL, had high porosity, without the support of natural components, their impact on angiogenesis was limited [21].

In summary, CS and CS/AG combinations generally promoted slight increases in various vascular parameters compared to the control, while AG alone had minimal effects and showed varied results depending on the specific parameter and time point. CS alone demonstrated the most consistent proangiogenic effects, enhancing both vessel length and the number of branching points. These findings suggest the potential application of CS and CS/AG materials in therapeutic angiogenesis, warranting further investigation into their mechanisms and long-term effects on vascular development.

#### 2.2.5. Wound Healing

An animal model is an essential element in biomedical research. Due to their genetic, physiological, and pathological similarities to humans, mice are helpful in preclinical studies. Experiments conducted on small rodents are essential for evaluating developed biologics, as they provide preliminary information about interactions with living organisms, specifically regarding biocompatibility and therapeutic efficacy. Furthermore, studies on animal models complement earlier biological research on proliferation, cytotoxicity, or cell migration. Moreover, the results from studies conducted on animal models constitute the first step that allows the conduct of clinical trials, which are decisive for confirming the utility of scaffolds in medical applications.

In this experiment, the effect of the CS/AG composition and its components, namely CS and AG applied alone, on wound healing was evaluated in a model of excisional dorsal skin injury in mice. CO_2_-saturated water (Water CO_2_) was applied to wounds in control mice. According to the literature, CS accelerates wound healing by stimulating immune cells like macrophages and fibroblasts, which shortens the inflammatory phase and promotes tissue proliferation. Additionally, CS enhances granulation tissue formation and angiogenesis, aiding collagen deposition for skin repair [9,10]. Therefore, this study aimed to determine whether the CS/AG composition accelerates wound healing, and specifically if the addition of agarose affects the efficacy of this process.

**Figure 8:**
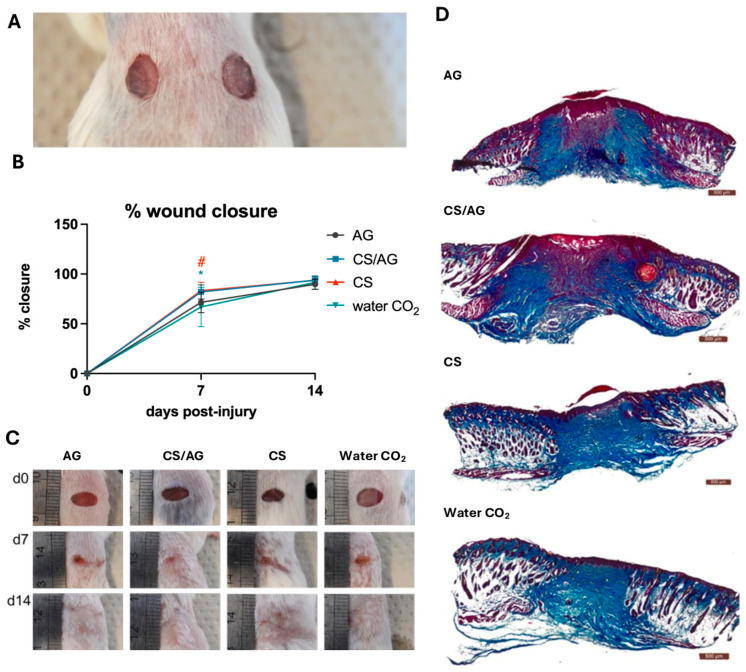
Effects of tested hydrogels on skin wound healing in mice. (**A**) Example photo of hydrogel discs applied on dorsal skin wounds; (**B**) mean percentage of wound closure; error bars represent SD, *n* = 12—a number of wounds representing 6 mice in each group; statistical significance relative to Water CO_2_ control was determined using the two-tailed Mann–Whitney test and denoted with an asterisk “*” for CS/AG and a hash “#” for CS alone; (**C**) representative images of wounds at 0, d7, and d14; (**D**) representative skin sections were collected on day 14 post-injury and stained with Masson trichrome to visualise tissue architecture: red—muscles and keratin; blue—collagen; pink—cytoplasm; brown—nuclei. The samples are indicated as follows: CS/AG—chitosan–agarose composition, CS—chitosan alone; AG—agarose alone; Water CO_2_—carbon dioxide-saturated water.

CS/AG and CS dressings applied as 6 mm hydrogel discs onto wounds (Figure 8A) significantly accelerated wound closure, resulting in 82.0 and 83.2% closure, respectively, on day 7 post-injury compared to the control (66.9%) (Figure 8B,C). On day 14 post-injury, wound closure in mice treated with each of three types of hydrogel dressings and in the controls reached approximately 90%. In the case of AG, the pace of wound closure was similar to that of the control group (Water CO_2_). On day 14 post-injury, wound closure equalled in all tested groups, including the controls, reaching approximately 90%. No complications in the wounds were observed, thus confirming the biocompatibility of the applied dressings. Histological examination (Figure 8D) indicated typical wound healing in all groups (Figure 8D).

It is crucial not only to concentrate on CS but also to apply it to the skin. The CS hydrogel rapidly releases CO_2_ and dries quickly. The AG in CS/AG allows the maintenance of a moist environment in contact with the wound, providing adequate moisture and protection against drying. This may be because AG creates a more stable and durable scaffold that better mimics the natural tissue environment. As a result, CS/AG dressings can combine chitosan’s biological activities with agarose’s physicochemical properties, promoting the maintenance of a moist microenvironment.

## 3. Materials and Methods

### 3.1. Materials

Chitosan—CS (MMW, DD > 75%), agarose—AG (low EEO, 34.5–37.5 °C gel point), phosphate-buffer saline (PBS) sodium hydroxide, and glycolic acid were purchased from Merck (Darmstadt, Germany). The carbon dioxide used to saturate the CS precipitate was derived from Linde Gaz Polska Sp. z o. o. (Gdańsk, Poland). Immortalised human keratinocyte HaCaT cells were purchased from the German Cancer Research Center (DKFZ, Heidelberg, Germany). The human skin fibroblast cell line 46BR.1N was obtained from the European Collection of Authenticated Cell Cultures (ECACC, Sigma Aldrich, St. Louis, MO, USA). The 3-(4,5-dimethylthiazol-2-yl)-2,5-diphenyltetrazolium bromide (MTT), DMEM, antibiotics, propidium iodide, calcein, and supplements necessary for cell culture were obtained from Sigma-Aldrich (St. Louis, MO, USA). Wound healing assay kits were obtained from Ibidi GmbH (Darmstadt, Germany). MilliQ water was used to prepare all aqueous solutions. All other reagents were of analytical grade or higher. The experiments on mice were conducted in the Tri-City Academic Laboratory Animal Centre of the Medical University of Gdańsk, where the animals were bred and maintained. 3,5-dinitrosalicylic acid, glucose, Resazurin sodium salt, methylene blue, Brilliant Green, Acid Fuchsin, and Nigrosin were purchased from Merck (Germany).

### 3.2. Hydrogel Preparation

**Figure 9:**
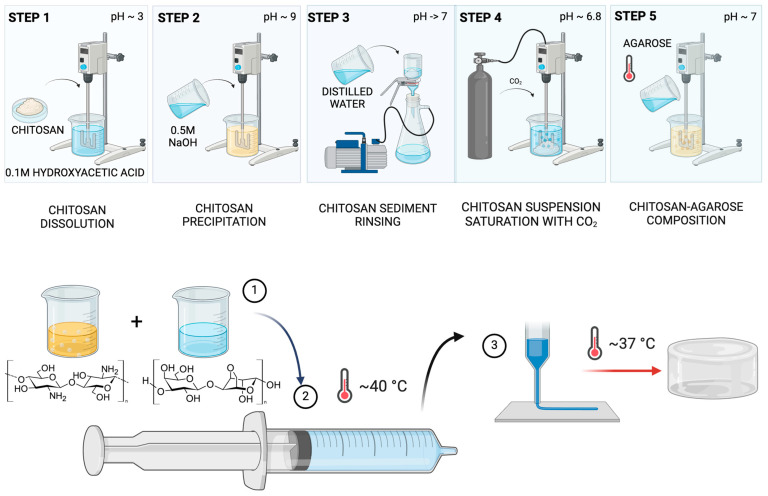
Schematic representation of the preparation of the chitosan–agarose composite using the carbon dioxide saturation method for chitosan processing. Created with Biorender.com.

The composition based on chitosan (CS) and agarose (AG), as well as their individual solutions, was prepared according to the methodology described by Banach-Kopeć et al., 2024 [22] (Figure 9). To summarise, a 1% CS solution with an average molecular weight was prepared using an innovative carbon dioxide saturation method. Meanwhile, a 1% AG solution was prepared in distilled water at 80 °C while stirring at 200 RPM (RA 2020, Heidolph Instruments GmbH & Co. KG, Kelheim, Germany). The CS/AG-based compositions were prepared by cooling the hot AG solution to 45 °C and immediately combining it with the CS solution in various weight ratios using a mechanical stirrer at a speed of 300 revolutions per minute for 2 min (BIOMIXBMX-10, Gdańsk, Poland). To evaluate the rheological properties, the temperature of the mixture was maintained at 40 °C to prevent gelation.

To standardise the water content of the CS/AG hydrogel membranes, it was necessary to initially reduce the water content and then replenish it through conditioning. This process ensured that the samples were consistent for repeatable testing. The membranes were cast onto 90 mm Petri dishes, dried at 25 °C for 48 h, and then incubated in distilled water for 24 h before measurement, resulting in a thickness of 0.4 mm. Samples for angiogenesis assessment were prepared in the same way, with the difference that they were incubated in a 0.9% saline solution. For the evaluation of wound closure, CS/AG and AG samples were cast onto 90 mm Petri dishes, and after gelation, 6 mm diameter discs were cut using a biopsy punch.

To conduct biological safety tests and assess the system’s impact on cell migration, samples of CS, AG, and their composites were frozen at −80 °C and then freeze-dried (Christ Alpha 12–4 LD Plus, Osterode am Harz, Germany). According to the standards of the MTT test and the Ibidi wound healing assay methodology, it is necessary to prepare extracts of the tested materials. To ensure the most rigorous conditions and achieve the highest possible concentrations of the tested extracts, the samples were freeze-dried rather than preparing extracts from their solutions. This approach is critical because, during the bioprinting process, the printed scaffolds are placed in the medium only after the process is complete, which can lead to significant water loss, especially in the first printed layers.

### 3.3. Physiochemical Characterisation of Chitosan–Agarose Composition

#### 3.3.1. Rheological Properties Assessment

The rheological properties of the CS and AG solutions and their composites were measured using an oscillating rheometer Anton Paar series MCR 302e (Anton Paar, Warsaw, Poland). A plate–plate system with a 25 mm diameter and 1 mm gap was used for the measurements. The impact of the shear rate on the deformation of the samples was conducted at a temperature of 37 °C and the method of a three-interval thixotropy test was applied (three-interval thixotropy test (3ITT) oscillations–rotations–oscillations). In the middle interval, the sample was deformed at a constant shear rate (1000 1/s). In the initial and final interval, the sample was tested through oscillatory shear at a constant deformation amplitude γ = 1% and angular frequency ω = 5 rad/s in order to establish the properties before and after the rotational deformation process.

#### 3.3.2. Permeability Assessment

A modified Kirby–Bauer method was applied to study permeability in AG and CS hydrogels [23]. This modification involved the use of dyes with varying molecular weights. Initially, 1% AG and CS solutions and their compositions were prepared, then spread on Petri dishes and left to gel. For the CS solutions, this process took 48 h to allow the release of carbon dioxide necessary for gelling. After the completion of the gelling process, 100 mM solutions of dyes with molecular weights ranging from 251 to 616 g/mol were prepared. In the next step, holes with a diameter of 6 mm were cut in the gelled samples using a biopsy punch. A total of 0.150 mL of the respective dye solution was applied to each of these holes. The samples were incubated at a temperature of 25 °C. The diameters of the dye spots were measured with a calliper at regular time intervals, which allowed for the assessment of the degree of diffusion of the dyes in the hydrogel matrices.

To assess glucose permeability, the method described by Reys and co-workers was used with slight modifications [15]. A laboratory setup was configured in which a round-bottomed three-necked flask was used as an acceptor. To this acceptor, a funnel and a metal filter from a filtration system under vacuum were connected to form the receptor part of the apparatus. The flask was placed in a temperature-controlled, i.e., 37 °C, water bath. Hydrogel membranes based on CS, AG, and their composites with a thickness of 0.4 mm were placed in the receptor part of the apparatus, stabilised with a stainless steel clamp. Before measurement, the samples were incubated for 24 h in distilled water. The round-bottom flask was then filled with distilled water and the donor space with a 10% glucose solution. At fixed time points, 2 mL samples were taken from the acceptor chamber, each time topped up with an equal amount of fresh water. The experiments were carried out with continuous stirring of the contents of the round-bottom flask using a magnetic rod to eliminate boundary layer effects that would interfere with the measurement. A colourimetric method for the determination of reducing sugars by reaction with 3,5-dinitrosalicylic acid (DNS method) was used to analyse the concentration of glucose that permeated the membrane into the round-bottom chamber. The measurement was performed at 540 nm using a Mettler UV5 spectrophotometer.

### 3.4. Biological Properties of Chitosan–Agarose Composition Assessment

#### 3.4.1. Cell Culture Conditions

Immortalised human HaCaT keratinocytes (DKFZ, Heidelberg, Germany) [24,25] and the human dermal fibroblast cell line 46BR.1N (ECACC, Sigma Aldrich, St. Louis, MO, USA) were used in this study. Cells were routinely grown in culture flasks (growth surface area 25 cm^2^) in Dulbecco’s modified Eagle medium (DMEM, Sigma Aldrich, St. Louis, MO, USA) with 4500 mg/L of glucose, 584 mg/L of L-glutamine, sodium pyruvate, and sodium bicarbonate, supplemented with 10% FBS, 100 units/mL of penicillin, and 100 μg/mL of streptomycin (Sigma Aldrich, St. Louis, MO, USA) under a humidified atmosphere with 5% CO_2_ at 37 °C. The medium was changed every 2–3 days.

#### 3.4.2. MTT

AG, CS, and CS/AG composites were prepared in accordance with the ISO 10993-5:2009 standard. An appropriate amount of medium dedicated to 46BR.1N and HaCaT cells was added to a given composite weight (25 mg per 1 mL culture medium). Then, the prepared composites were placed in a CO_2_ incubator for 24 h. After this time, the cells of the 46BR.1N and HaCaT lines seeded in a 96-well plate were replaced with the medium corresponding to a given composite for stimulation in two concentrations: 100%—adding the full volume of undiluted supernatant from previously prepared composites; and 50%—half the volume of the supernatant from a given composite diluted with DMEM HG. The cells were incubated for 24 h. After this time, the MTT test was performed in accordance with the manufacturer’s instructions. MTT solution (5 mg/mL) was added to each well. After 3 h, the medium was removed, and isopropanol (6 mM) was added to the cells. Then, the plates with the cells were shaken for 30 min, and a spectrophotometric reading was performed (Synergy LX, Biotek, Winooski, VT, USA) at a wavelength of 575 nm.

#### 3.4.3. Cell Viability Assessment

To investigate the effectiveness of the newly developed bioink in maintaining cellular viability, an experiment was conducted using a mixture of CS (1% *w*/*v*) of medium molecular weight and AG (1% *w*/*v*). These components were combined in equal proportions and incubated at 37 °C to prevent the composition from gelling. Cells, at a concentration of 1 × 10^6^ cells/mL, were integrated with the prepared bioink and then applied to 24-well culture plates using a syringe, forming 3D structures. After the scaffolding gelled below 37 °C, the wells were filled with the appropriate medium and placed in an incubator (37 °C, 5% CO_2_).

Selective staining was performed to colour live cells green and dead cells red to assess cellular viability in the produced structures. After removing the medium, the scaffolds were cleansed with phosphate-buffered saline (PBS). Then, 3 mL of PBS solution with the addition of calcein-AM (8 µM) and propidium iodide (2 µg/mL) was applied into each well. The scaffolds were incubated with the dyes in darkness at a constant temperature of 37 °C in a 5% CO_2_ environment for 45 min. After the incubation, the staining solution was removed, and fluorescence microscopy observations were made (Nikon Eclipse TE300, Nikon, Tokyo, Japan). The use of CellProfiler 4.2.5 software enabled detailed analysis of the obtained images and quantification of live and dead cells.

#### 3.4.4. Wound Healing Ibidi

The study on the impact of CS in combination with AG on the migratory capacity of cells after 24 h was conducted using Ibidi culture inserts with a predefined cell-free area, crucial for research in wound healing and cell migration. For the experiment, cells were seeded in the inserts at a concentration of 20,000 per well, utilising DMEM enriched with 10% FBS. After 24 h, the medium was replaced with serum-free DMEM, and cellular growth was inhibited by adding mitomycin C (5 μg/mL) for 2 h. Following this procedure, the medium was changed, and the cells were subjected to the extracts of the tested CS, AG, and CS/AG compositions. After another 24 h, the cells were fixed using 3.7% paraformaldehyde and then stained with a 0.05% solution of crystal violet. The results were evaluated using a Leica DMIL LED inverted microscope and GRAPHAX software.

#### 3.4.5. Angiogenesis Assessment

Fertilised chicken eggs were obtained from a local provider (EKO ELITA, Jawory, Poland) and incubated horizontally with intermittent rotation at 37 °C and 65% humidity for 3 days. Subsequently, the eggshells were cracked open, and the embryos were transferred into sterile containers for further incubation at 37 °C with 0.5% CO_2_ for 5 days. On day 8, approximately 5 mm square pieces of various materials (CS, AG, CS/AG, or Whatman filter paper) were placed on the chorioallantoic membrane (CAM). Prior to placement, all materials were UV-sterilised and rinsed with 0.9% NaCl solution. The materials were incubated on the CAM for up to 72 h. The integration of the materials with the CAM was photographed every 24 h using a stereomicroscope attached to a digital camera (Olympus Stemi 2000-C). Angiogenesis around the materials was quantified using the AngioTool 0.6 software on the IKOSA platform. On embryonic development day (EDD) 12, the embryos were humanely sacrificed. In the European Union, the CAM assay is not legally classified as an animal experiment and, therefore, does not require ethical approval (EU Directive 2010/63/EU and European Commission 2021/2784 (RSP)).

#### 3.4.6. Wound Healing Model in Mice

A full-thickness excisional skin injury model in mice was used to investigate the effects of hydrogel compositions on wound healing. The mice, 8–10-week-old females of the BALB/c strain, were purchased from the Tri-City Academic Laboratory Animal Centre of the Medical University of Gdańsk, where they were maintained. All animal experiments were performed in compliance with the ARRIVE guidelines. The experiment protocol received approval from the Local Ethical Committee in Bydgoszcz (approval number 51/2020).

Mice were anaesthetised with isoflurane inhalations at 2–5% concentration. Before starting the experiment, the animals’ backs were shaved and disinfected with Octanisept. Then, the skin on the back was folded and lifted towards the head and tail along the spinal line and biopsied using 6.0 mm diameter punches to create two symmetrical wounds. Before the application of the tested samples, mice were randomly divided into therapeutic and control groups, each consisting of six animals. Then, 2 mm thick discs of 6 mm diameter prepared from CS/AG and AG hydrogels were placed onto the wounds. The wounds in the control mice were treated with 10 µL of carbon dioxide-saturated water. The wounds were then covered with transparent Tegaderm^TM^ dressing (25 × 25 mm) and fastened with a self-adhesive bandage wrapped around the animal torso. After 7 days, the dressings and test preparations were replaced to avoid damaging the newly formed skin layer. After 14 days, the dressings were removed. A ruler was placed beside the wound area to measure the wound area, and photographs were taken weekly. Wound sizes were calculated using ImageJ 1.52a/Java 1.8.0_112 [26].

#### 3.4.7. Tissue Isolation for Histological Analyses

After collection, the ears were preserved in 4% formalin buffered with PBS. The tissues were then transferred to a 15% sucrose in PBS for 24 h and then to a 30% sucrose solution in PBS for another 24 h. Then, the tissues were sectioned to 10 μm thickness on the cryostat Leica CM1520 and stained with Masson’s trichrome. Image acquisition was performed with a Leica DM IL microscope at 40× magnification.

### 3.5. Statistical Analysis

All data reported were based on the means of three replicates (*n* = 3), except for Ibidi wound healing tests, which were performed in repetitions for three independent tests (*n* = 15), and wound healing tests in mice, where the results represented means for 12 wounds in 6 mice (*n* = 12). Experimental results were expressed as mean ± standard deviation (SD). Statistical analysis of biocompatibility of cell culture results was performed with one-way ANOVA. The differences were considered to be statistically significant at *p* < 0.05. Prism 10 V10.0.3 (GraphPad software, Boston, MA, USA) was used for the statistical computations.

## 4. Summary and Conclusions

This research continues the investigation into an innovative thermosensitive bioink composed of a medium-molecular-weight chitosan hydrogel formed by CO_2_ saturation and an agarose hydrogel responsible for gelling. After meeting the bioink acceptance criteria, we conducted further characterisation to assess detailed flow characteristics during simulated syringe extrusion in 3D printing. An ORO test revealed that the three-dimensional structure of the CS/AG composite rapidly recovers after extrusion, unlike its components. Studies on the diffusion rates of dyes through CS/AG membranes showed that both polymer proportions and molecular weight significantly affect diffusion. Glucose permeability tests, using glucose as a model compound mimicking nutrient diffusion, confirmed effective diffusion through various CS/AG compositions, highlighting its regenerative potential.

Further studies evaluating biological safety and activity used MTT tests on selected skin cell lines (46BR.1N fibroblasts and HaCaT keratinocytes) intended as key components of the bioink. These tests confirmed the biological safety of CS, AG, and their composites in line with ISO 10993-5. The tested samples showed high cell viability, with values ranging from 85% to 112% for 46BR.1N and 90% to 137% for HaCaT cells, indicating a favourable safety profile. Additionally, it was shown that the CS/AG composition favours HaCaT cell migration, a promising finding for regenerative applications. However, 46BR.1N cells displayed moderate pro-migratory effects, suggesting the need for further optimisation to meet specific cell requirements.

In addition to cytotoxicity tests conducted after a 24 h incubation, the long-term survival of cells within the CS/AG composition was monitored. Cell survival rates for HaCaT and 46BR.1N fibroblasts after 2, 7, and 14 days confirmed that the biomaterials not only support cell proliferation but are also biocompatible. High survival rates, particularly at day 14, demonstrated the crucial role of chitosan in enhancing cell survival over time.

Based on studies using L929, HaCaT, and 46BR.1N cell lines, along with preliminary evaluations of printability and functionality, a detailed analysis of the proangiogenic potential of CS/AG biomaterials was performed. Both CS and CS/AG composites exhibited superior proangiogenic properties compared to pure AG and the control. The absence of angiogenesis inhibition further emphasises the potential of the CS/AG composition for tissue engineering applications.

The confirmed biological safety and favourable properties of CS/AG enabled the transition to animal model studies, a critical step in assessing the effectiveness of biological therapeutic systems. Studies conducted on mice showed that CS/AG and CS dressings accelerated skin wound closure, demonstrating strong potential for future use in regenerative medicine.

## 5. Further Action

In our most recent studies, we were able to confirm the positive impact of the CS/AG composition on many critical biological parameters, opening up new possibilities for its use in tissue engineering. The next step will be to investigate the impact of the addition of time-releasing bioactive peptides on migratory effects and the wound healing process, as well as the possibility of creating a vascularised scaffold. Additionally, future research stages will include designing the geometry of the scaffold, optimising the arrangement of layers and cells, and determining the porosity of the scaffold to assess its permeability. Such a comprehensive approach will allow for the further customisation of bioink properties, which is crucial for its future clinical applications.

## Data Availability

Data are contained within the article.
